# Biochemical Characterization of Two Clinically-Relevant Human Fumarase Variants Defective for Oligomerization

**DOI:** 10.2174/1874091X01812010001

**Published:** 2018-01-29

**Authors:** Artemisa Bulku, Todd M. Weaver, Melanie B. Berkmen

**Affiliations:** 1 Department of Chemistry and Biochemistry, Suffolk University, 8 Ashburton Place, Boston, MA, USA; 2 Department of Chemistry and Biochemistry, University of Wisconsin-La Crosse, La Crosse, WI, USA

**Keywords:** Fumarase, Fumarate hydratase, Fumarate hydratase deficiency, Fumaric aciduria, Hereditary leiomyomatosis, Renal cell cancer

## Abstract

**Background:**

Fumarase, a significant enzyme of energy metabolism, catalyzes the reversible hydration of fumarate to L-malate. Mutations in the *FH* gene, encoding human fumarase, are associated with fumarate hydratase deficiency (FHD) and hereditary leiomyomatosis and renal cell cancer (HLRCC). Fumarase assembles into a homotetramer, with four active sites. Interestingly, residues from three of the four subunits within the homotetramer comprise each active site. Hence, any mutation affecting oligomerization is predicted to disrupt enzyme activity.

**Methods:**

We constructed two variants of hexahistidine-tagged human recombinant fumarase, A308T and H318Y, associated with FHD and HLRCC, respectively. Both Ala308 and His318 lie within the fumarase intersubunit interface. We purified unmodified human fumarase and the two variants, and analyzed their enzymatic activities and oligomerization states *in vitro*.

**Results:**

Both variants showed severely diminished fumarase activity. Steady-state kinetic analysis demonstrated that the variants were largely defective due to decreased turnover rate, while displaying K_m_ values for L-malate similar to unmodified human recombinant fumarase. Blue native polyacrylamide gel electrophoresis and gel filtration experiments revealed that each variant had an altered oligomerization state, largely forming homodimers rather than homotetramers.

**Conclusion:**

We conclude that A308T and H318Y render human fumarase enzymatically inactive via defective oligomerization. Therefore, some forms of FHD and HLRCC can be linked to improperly folded quaternary structure.

## 1 INTRODUCTION

Fumarase (EC 4.2.1.2), or fumarate hydratase (FH), catalyzes the reversible hydration of fumarate to L-malate [1, 2]. Fumarase enzymes are found in all three domains of life and are strikingly conserved. For example, human and *Escherichia coli* fumarase share 60% amino acid sequence identity [3] and human fumarase can complement a *fum1* deletion in yeast [4]. Moreover, the (GS^365^SxxPxK^371^xN^373^) signature sequence can be used to identify fumarase family members [5]. All amino acid numbering within this manuscript is based upon the human fumarase protein sequence, P07954 [6]. The human fumarate hydratase gene (*FH)*, located on chromosome 1q42.1 [7], encodes both mitochondrial and cytosolic isoforms of fumarase [8]. Mitochondrial fumarase participates in the Krebs cycle, whereas cytosolic fumarase assists in the metabolism of fumarate, a by-product of amino acid metabolism and the urea cycle [9, 10].

Mutation of *FH* is associated with a rare metabolic disease known as fumarate hydratase deficiency (FHD) or fumaric aciduria (OMIM 606812; reviewed in [11-14]). FHD is inherited in an autosomal recessive manner with individuals exhibiting low levels of fumarase activity in fibroblasts or other cells, and high levels of fumaric acid in urine. Some symptoms of FHD include hypotonia, cerebral malformation and atrophy, seizures, failure to thrive, and developmental delay [15-18]. As therapeutic strategies are lacking, many individuals with FHD do not survive past early childhood.

Fumarase also functions as a tumor suppressor, where certain germline loss-of-function mutations in *FH* predispose individuals to a variety of tumors and cancers [19-24]. Mutations in *FH* are closely associated with multiple cutaneous and uterine leiomyomatosis syndrome (MCUL), manifested by the development of benign smooth tumors in the skin and uterus. This disease is now referred to as hereditary leiomyomatosis and renal cell cancer (HLRCC; OMIM 150800), due to more recent recognition of the associated increased risk for kidney cancer. More recently, *FH* mutations have been linked to paragangliomas and pheochromocytomas, tumors in the neuroendocrine tissues and the adrenal medulla, respectively [25, 26] as well as bladder, breast [27], and testicular cancers [28]. Several mechanisms have been proposed to explain how FH functions as a tumor suppressor [4, 29-37].

**Fig. (1):**
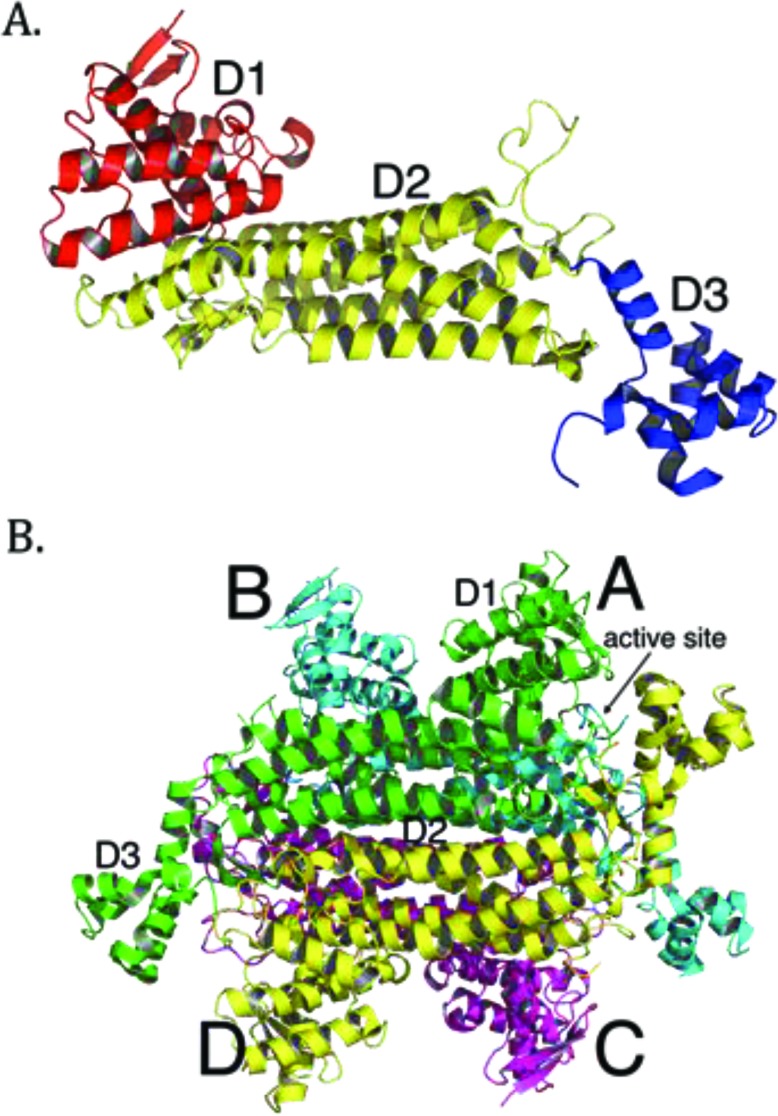
Tertiary and quaternary structure of human fumarase (PDB ID: 3E04 [40]). (**A**) A single subunit of human fumarase has been colored-coded by domain: domain 1 (D1) – red, domain 2 (D2) – yellow, and domain 3 (D3) – blue. (**B**) The human fumarase homotetramer has been colored by chain, with subunits labeled as A, B, C, or D. The all α-helical central domain, D2, forms the major intersubunit interface upon oligomerization. D1 and D3 domains lie at the corners of the homotetramer and outline the entry point to the four independent fumarase active sites.

More than 100 mutations in the *FH* gene associated with disease have been reported so far, with the majority being missense mutations [38]. Most clinically relevant missense mutations affect residues that are evolutionarily conserved [39, 40]. Other mutations, such as deletions or splice-site mutations, often result in omission of whole segments of the protein and thus cause severe disruptions in conformation of the protein [25, 41, 42]. Overall, mutations in *FH* seem to cause structural changes to the enzyme that eliminate or compromise enzymatic function. Many efforts have been made to link particular mutations to the structure and function of fumarase. The crystal structures of *E. coli* fumarase C (FumC) [43], yeast fumarase [44], and human fumarase [40] are highly similar, consistent with their high sequence identity. Fumarase is a homotetramer, with each fumarase subunit constructed from three domains termed D1, D2, and D3 (PDB ID: 3E04 [40]; Fig. (**1A**). The central domain, D2, facilitates tetramerization (Fig. **1B**). Each of the four independent active sites, deduced by co-crystals with inhibitors and through biochemical analyses of point mutants [43, 45-47], are located between domains D1 and D3 at the four corners of the enzyme (Fig. **1B**).

**Fig. (2):**
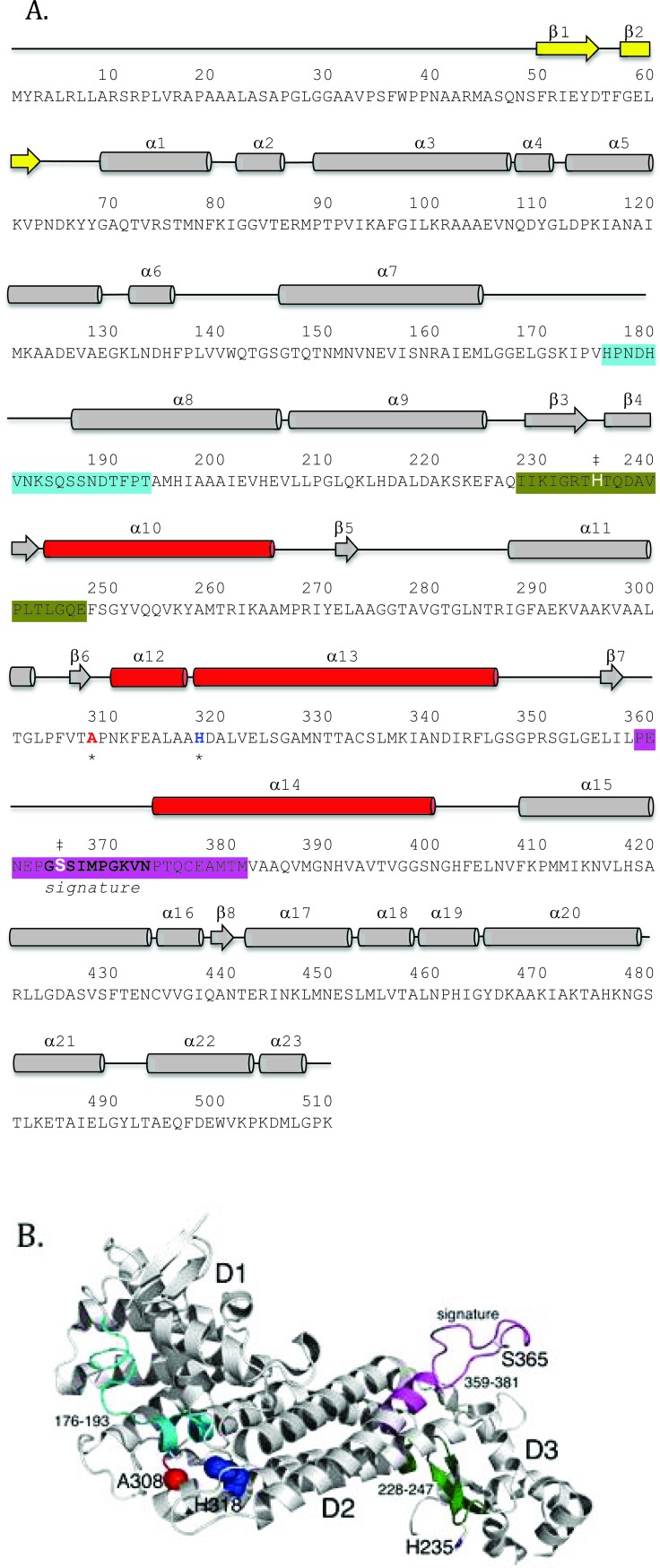
Primary and tertiary structure representations of the fumarase family highly conserved regions. (**A**) The primary sequence of human fumarase (P07954: FUMH_HUMAN) has been annotated with secondary structural elements, fumarase family highly conserved regions, active site residues H235 and S365, and the clinically observed variant (A308 and H318) locations. The three regions of high conservation observed within the fumarase family have been colored accordingly: cyan (region 1 residues 176-193), dark green (region 2 residues 228-247), and magenta (region 3 residues 359-381). Region 3 also defines the “signature sequence” for the fumarase super-family. Regions 1 and 3 form the majority of the substrate-binding site, while regions 2 and 3 contribute the catalytic groups H235 and S365 (denoted by the double dagger symbol). Additionally, A308 and H318 have been denoted with an asterisk and colored red and blue, respectively. (**B**) The tertiary structure of the fumarase monomer (PDB ID: 3E04 [40]) emphasizes the three-dimensional juxtaposition of the highly conserved regions within the fumarase family. The tertiary structure has been colored and annotated similar to panel (**A**). The three-dimensional view of the fumarase monomer emphasizes (1) the spatial distance between the highly conserved regions (1-3) and (2) the locations of A308 and H318 near α-helices 12 and 13 of D2.

Structurally, each human fumarase subunit harbors twenty-three α-helices and eight β-strands (Fig. **2A**) [40]. Amino acid alignments conducted on fumarase and other superfamily members, including aspartase, arginosuccinate lyase, adenylosuccinate lyase, and carboxy *cis*-*cis* muconate lactonizing enzyme, have identified three regions of significant conservation (Fig. **2A**) [5]. From a three-dimensional perspective, these three conserved areas lie in disparate areas of the monomeric structure (Fig. **2B**). However, upon tetramerization these regions coalesce to form the four active sites. More specifically, residues 176-193, 228-247 and 359-381, donated from three distinct subunits, form each fumarase active site. The first and third conserved regions construct the majority of the substrate-binding site, while the second and third regions donate the catalytic groups H235 (catalytic acid) and S365 (catalytic base), respectively (Fig. **2B**) [1].

More than 50 *FH* mutations have been mapped onto the crystal structure of fumarase [39-41, 48]. Many mutations are located in or near the active site, and thus likely affect enzymatic activity directly. A few mutations affecting the active site of *E. coli* FumC have been shown to decrease activity of the purified enzyme [3, 45, 46]. In principle, a mutation that affects oligomerization should affect enzymatic activity as each of the four active sites in the enzyme is composed of residues donated from three of the four subunits of the homotetramer (Figs. **2A** and **2B**) [46]. A few reports have suggested that fumarase is only active as a tetramer [49, 50]. The cited evidence pertains to experiments that showed that fumarase treated with guanidine hydrochloride, urea, or organic solvents becomes both monomeric and enzymatically inactive. However, these reagents would also denature the protein, which could also explain the observed loss of activity.

Another analysis of a very common HLRCC FH variant (R190H) suggests that tetramerization is required for activity [51]. Over-expression of FH (R190H) in cells containing wild-type FH causes a dominant-negative loss of fumarase activity and loss of FH tetramers. R190 forms an ion pair with a glutamic acid residue from the same subunit and is located on the section that contributes the active site histidine. Two more interesting structural notes follow: (1) R190 also forms a hydrogen bond with the carbonyl oxygen of a serine residue from a neighboring subunit and (2) the hydrogen bond shared between R190 and the glutamic acid is located within a hydrophobic region (residues donated from 3 of 4 subunits). The hydrophobic region would serve to strengthen the hydrogen bond shared between these two charged residues. So, it is not surprising that the R190H would be deleterious to fumarase structure and function.

In this study, we purified and characterized the enzymatic activities and oligomerization states of recombinant human fumarase (HsFH) and two disease variants of the enzyme (A308T and H318Y). The A308T mutation (c.922G>A in exon 7) was previously identified in a patient with FHD that presented with hypotonia, microcephaly, and slow development [17, 52]. The other mutation investigated, H318Y (c.952A>T in exon 7), has been found in several patients diagnosed with HLRCC or MCUL [53-55]. Both alanine 308 and histidine 318 are located in the central D2 domain (Figs. **2A** and **B**) [43] and were previously hypothesized to disrupt intersubunit interactions based on their locations in the crystal structure of human fumarase (PDB ID: 3E04) [40]. Our biochemical analyses of the variants indicate that both are defective for enzymatic activity and oligomerization. Our data provide new biochemical insights into how mutations in *FH* lead to disease and provide support for the model that a functional active site requires tetramer formation. Throughout the manuscript, we refer to unmodified recombinant human fumarase as HsFH, while the two variants are identified as A308T and H318Y, respectively.

## 2 MATERIALS AND METHODS

### 2.1 Site-Directed Mutagenesis

The parental plasmid pET28α-HsFH [56], which expresses *E. coli* codon-optimized human fumarase domain (residues 44-510) with an N-terminal His6-tag, was generously provided by Maria Cristina Nonato and Ricardo Augusto Pereira de Padua (Universidade de Sao Paulo, Brazil). Site-directed mutagenesis was performed using the Q5 mutagenesis kit (New England Biolabs), according to manufacturer’s instructions. Plasmid pMMB1788 was constructed to encode *His_6_-HsFH (H318Y);* H318Y was previously reported as H275Y in the older numbering system for fumarase [38], which omitted the cleaved N-terminus. Plasmid pMMB1789 was constructed to contain *His_6_-HsFH(A308T)*; A308T was previously reported as A265T. After site-directed mutagenesis, each entire *FH* gene was sequenced to confirm successful incorporation of the designed alteration.

### 2.2 Protein Purification

HsFH and the two variants (A308T and H318Y) were overexpressed and purified largely following previously reported procedures [56]. For overexpression, the plasmids were transformed fresh into T7 Express Competent *E. coli* (New England Biolabs), selecting on LB-kanamycin (25 µg/mL). Cells were grown overnight in LB at 37°C and then diluted 100-fold the next morning into fresh LB-kanamycin (25 µg/mL). At OD600 ~0.5, cells were induced with 1 µM IPTG overnight at 19°C. Cells were harvested the following day via centrifugation.

Cell pellets were suspended in lysis buffer (50 mM sodium phosphate pH 7.3, 150 mM NaCl, 5 mM β-mercaptoethanol and 0.5 mM PMSF). Cells were lysed with a sonicator (Misonix XL 2000) or LM20 microfluidizer (Siemens). The soluble fractions were collected after centrifugation at 14,000 rpm for 10 minutes and incubated with His-Select nickel affinity gel (Sigma) or Ni Sepharose 6 Fast flow (GE Healthcare) for 30 minutes at 4°C. After three washes with wash buffer (lysis buffer containing 20 mM imidazole), HsFH and the two variants were eluted with elution buffer (lysis buffer containing 250 mM imidazole). The eluates were first dialyzed against dialysis buffer A (50 mM phosphate pH 7.3, 150 mM NaCl, 10% glycerol and 1 mM DTT) and then dialyzed against dialysis buffer B (50 mM phosphate pH 7.3, 150 mM NaCl, 50% glycerol and 1 mM DTT). Samples collected during the purification were analyzed by sodium dodecyl sulfate polyacrylamide gel electrophoresis (SDS-PAGE) [57] using a 7.5% polyacrylamide gel (Bio-Rad) and running buffer (0.025 M Tris, 0.192 M glycine, and 0.1% SDS). *E. coli* fumarase C (FumC) and the E315Q variant were expressed and purified as previously noted [3].

### 2.3 Kinetic Analysis

To measure the enzymatic activity of HsFH, fumarase assays were performed as previously reported [57]. Fumarase activity was determined by measuring the conversion of L-malate to fumarate, monitoring the increase in absorbance at 250 nm. The concentration of fumarase was determined by absorbance at 280 nm using a theoretical extinction coefficient of 24,410 M^-1^ cm^-1^, obtained from the ExPASy [58]. Where necessary, protein was diluted with dialysis buffer B prior to analysis. Assays contained 0.001 mg/ml HsFH, 0.116 mg/mL H318Y, or 0.050 mg/mL A308T. Assays were performed in phosphate-buffered saline (PBS; 0.137M NaCl; 2.7 mM M KCl; 11.9 mM phosphate, pH 7.4) at room temperature (~23°C) in an accuScan GO plate reader (Fisher Scientific) using SkanIt software. Assays were started with the addition of L-malate (varied from 0.39 to 25 mM). The extinction coefficient of fumarate at 250 nm (1.43 mL µmol^-1^ cm^-1^) was experimentally determined under these conditions. The V_max_ and K_m_ were calculated using nonlinear regression for the Michaelis-Menten equation using Excel Solver (R^2^ > 0.99). The V_max_ and K_m_ reported are the averages obtained from four separate trials. The turnover number (k_cat_) was calculated per monomer.

### 2.4 Analytical Gel Filtration

Analytical gel filtration was performed using a Superdex 200 10/300 GL column (AKTA; GE Healthcare) equilibrated in dialysis buffer. The apparent molecular mass of fumarase was calculated based on a calibration curve produced using a mixture of proteins standards of different molecular masses (25, 158, 440 and 669 kDa) run on the same column with the same buffer. Each protein was analyzed at least three times.

### 2.5 Blue Native-PAGE (BN-PAGE)

For BN-PAGE analysis, protein concentration was determined using the Bradford assay [59], using Bovine Serum Albumin as a standard. BN-PAGE was performed as previously described [60, 61]. Samples were prepared by mixing purified HsFH, A308T, H318Y, FumC, and E315Q with BN-PAGE sample buffer (50 mM BisTris, 10% glycerol, 50 mM NaCl, 0.001% Ponceau S) and dialysis buffer when dilution was needed. Samples (5, 10, or 20 µg protein) were loaded into a 4-16% Bis-Tris Native PAGE gel (Fisher Scientific). Native PAGE running anode buffer (50 mM BisTris and 50 mM Tricine) and Native PAGE running cathode buffer (0.23 mM Coomassie G-250, 50 mM BisTris and 50 mM Tricine) were used. Gels were run at 4°C for 60min at 150 V and another 30 minutes at 250 V. Gels were fixed in fixing solution (40% methanol, and 10% acetic acid) and destained in 30% acetic acid prior to visualization.

## 3 RESULTS

### 3.1 Differential Positioning of A308 and H318 Within the Three Symmetry Related Fumarase Dimers

**Fig. (3):**
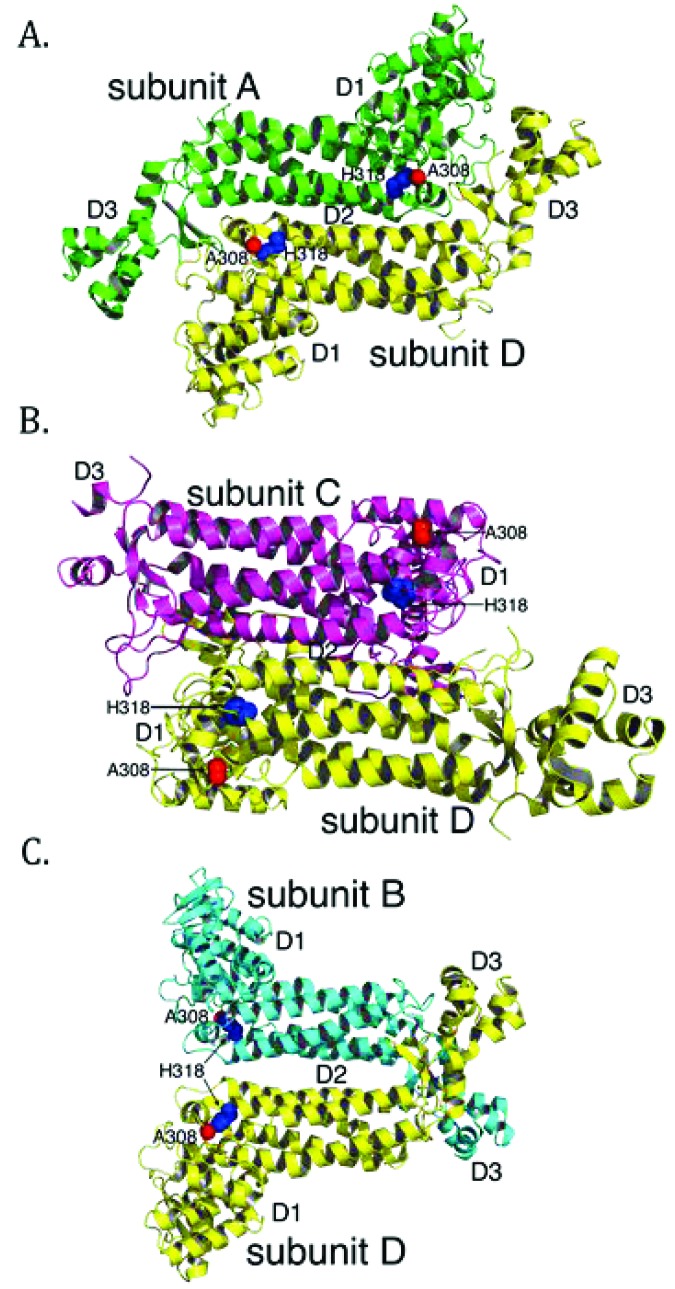
The positions of A308 and H318 differ within the three symmetry related dimer combinations of the human fumarase structure (PDB ID: 3E04 [40];). Three dimer interfaces derive from the fumarase homotetramer: (**A**) subunit A-subunit D (A-D), (**B**) subunit C-subunit D (C-D), and (**C**) subunit B-subunit D (B-D). The average calculated surface area across the three dimers is 3,000 (A-D), 2,300 (C-D) and 1,700 Å^2^ (B-D), respectively. Structurally, the intersubunit positions of A308 and H318 are nearest within the A-D dimer. The A-D dimer forms via interactions between residues donated by α-helices 12 and 13 of neighboring subunits. At the C-D dimer interface the positions of A308 and H318 are spatially removed. The B-D dimer shares the least amount of interacting surface area and the positions of A308 and H318 (within each monomer) are spatially distant. Monomers have been colored as depicted in Fig. **1B**.

To better understand the molecular basis of fumarase-related disease and the role of oligomerization with respect to fumarase activity, we analyzed two clinically relevant variants of human fumarase, A308T and H318Y. Although conserved from yeast to humans, residues A308 and H318 are outside of the three regions that contribute residues to the active site (Fig. **2A** and **2B**). Thus, these variants are unlikely to affect catalysis directly. However, A308 and H318 reside near and within α-helices 12 and 13, respectively. Helices 12 and 13 donate side chains involved in forming the three unique dimer interfaces: (1) subunit A-subunit D, (2) subunit B-subunit D, and (3) subunit C-subunit D (Fig. **3**). These dimers will be referenced as A-D, C-D, and B-D throughout the rest of the manuscript. Using PISA [62], we calculated that the three different dimers (A-D, C-D, and B-D) comprise interfaces of 3,000, 2,300, and 1,700 Å^2^ with associated free energy of interface formations of -41, -37, and -14 kcal/mol, respectively. Therefore, human fumarase variants (A308T and H318Y) in this region of α-helices 12 and 13 may indirectly diminish activity by directly affecting oligomerization, a requisite of fumarase function [43].

### 3.2 A308T and H318Y Affect Substrate Binding and Turnover Number Disparately

**Fig. (4):**
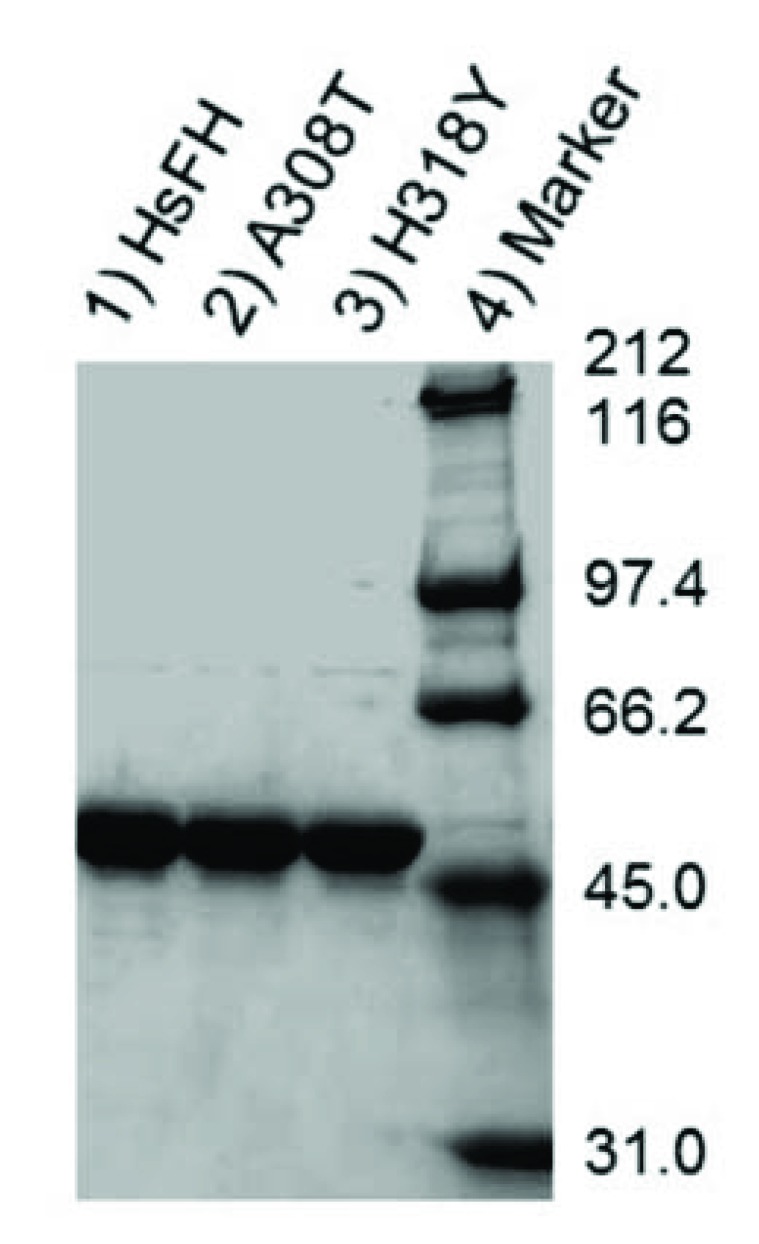
SDS-PAGE analysis of protein purity. HsFH (lane 1), A308T (lane 2), H318Y (lane 3), and protein molecular mass standards (lane 4).

**Fig. (5):**
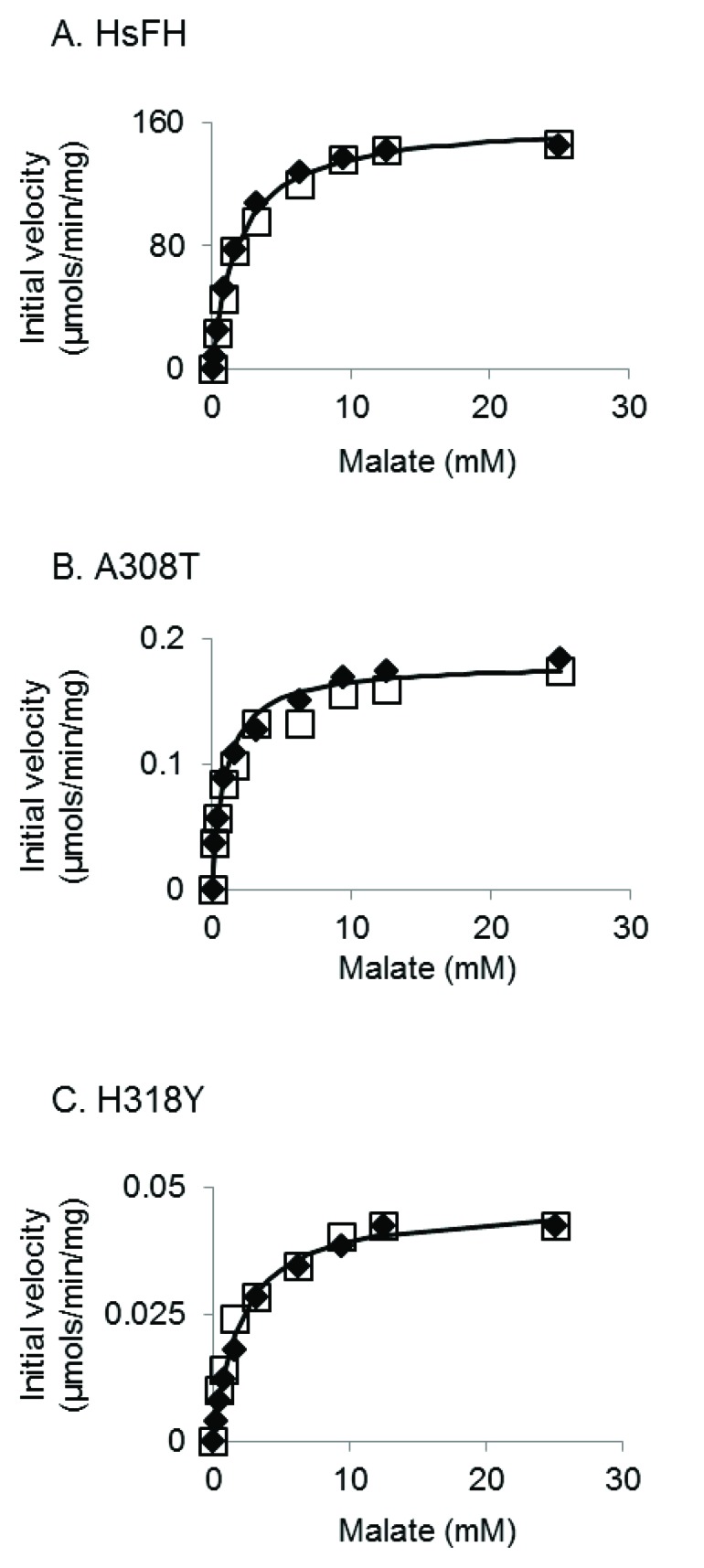
Representative kinetic plots of fumarase activity measured for: (**A**) HsFH, (**B**) A308T, and (**C**) H318Y. Initial velocities are plotted versus malate concentration and fitted using the Michaelis-Menten equation (solid lines). Each figure contains the data from two individual experiments (denoted as either open boxes or filled diamonds).

HsFH, A308T and H318Y were purified with an apparent molecular mass (53.8 kDa) (Fig. **4**), as expected [56]. Fumarase assays were used to verify that recombinant HsFH purified from *E. coli* follows Michaelis-Menten kinetic behavior. To our knowledge, no prior report has documented Michaelis-Menten kinetic values (K_m_ and V_max_) for purified human fumarase. During the Michaelis-Menten experimentation, fumarase activity was monitored spectrophotometrically via fumarate formation. HsFH was catalytically active and conformed to steady-state kinetic analysis (Fig. **5A**). Our assays revealed that HsFH has a V_max_ of 170 µmols/min/mg enzyme (Table **1**), nearly identical to that previously reported for the *E. coli* homolog FumC [3]. The turnover rate for recombinant HsFH is within two-fold of native pig heart fumarase [2]. HsFH affinity for L-malate (K_m_ = 1.9 mM; (Table **1**) is approximately two-fold lower than *E. coli* FumC [3] and about two-fold higher than that reported for pig heart fumarase [63]. We conclude that recombinant human fumarase, as purified from *E. coli,* is catalytically active. Thus, human fumarase does not require any human-specific post-translational modifications for basic enzymatic function. This is interesting to note since fumarase has been shown to be phosphorylated and modified in other ways within eukaryotic cells [32, 52].

We next conducted kinetic analysis of the variants A308T and H318Y (Figs. **5B** and **C**). We found that both variants were severely catalytically defective (Table **1**), primarily due to a reduction in (>900-fold) turnover rate (k_cat_). Both variants appeared to bind the substrate as well as HsFH, as indicated by their K_m_ values (Table **1**). Overall, the catalytic efficiencies for the A308T and H318Y mutants have been lowered ~500- and ~4000-fold, respectively. The large decreases in the turnover number presumably contribute to the previously observed disease states observed within affected individuals.

Interestingly, the fumarase activity of the A308T variant was previously measured in skin fibroblasts isolated from a patient homozygous for this mutation [52]. Relative to controls, the A308T variant was found to be ~20% active [52], which is on the very high end of values for FHD patients [14, 15, 17, 38, 39, 53-64] and an order of magnitude higher than what we found (Table **1**). There are a number of possible differences between their measurements and ours that could account for this discrepancy, such as differences in temperature, protein purity, and cell source of protein.

### 3.3 A308T and H318Y Shift the Homotetramer to Homodimer Equilibrium

Since alanine 308 and histidine 318 localize in α-helices 12 and 13 of D2, the fumarase tetramerization domain, we sought to determine whether the A308T and H318Y variants had altered oligomerization properties. A defect in intersubunit interactions could cause improper tetramer and active site assembly, resulting in diminished fumarase activity. We used two different non-denaturing methods to determine oligomerization status: (1) gel filtration and (2) blue native PAGE (BN-PAGE). Both methods indicated that the variants had profound defects in homotetramer formation.

We first used analytical gel filtration to analyze the oligomerization state of HsFH, A308T and H318Y. The bulk of HsFH eluted with an apparent molecular mass of ~210 kDa, consistent with the predicted mass of the homotetramer (215 kDa), and a small fraction of the protein eluted as higher order oligomers. Interestingly, purified *Heliobacter pylori* fumarase is also largely tetrameric with a small fraction of larger oligomers [65]. We did not detect monomeric or dimeric species of HsFH. In contrast, the majority of both variant proteins eluted aberrantly with apparent molecular masses of ~135 kDa. We suspect that these proteins are slightly irregularly shaped dimers, as a dimer would have a predicted mass of 108 kDa. We conclude that both amino acid substitutions caused improper homotetramer assembly leading to dimer formation as the major species.

**Fig. (6):**
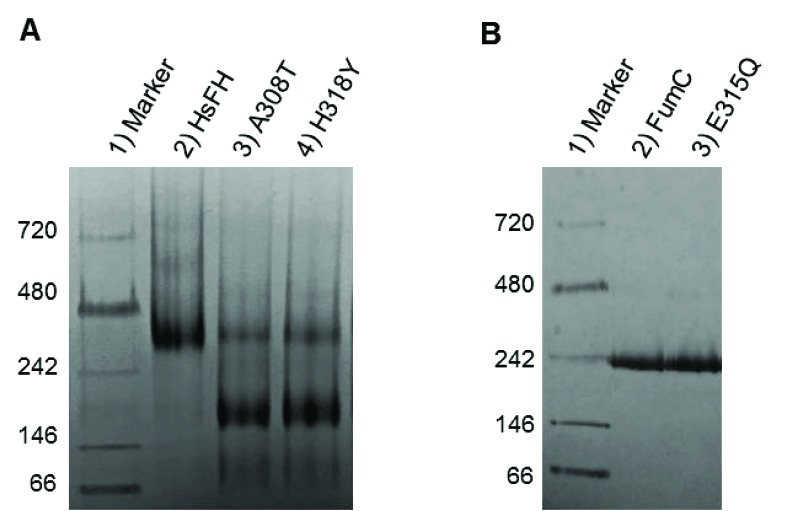
BN-PAGE analysis of purified fumarase variants (5 µg loaded). (**A**) Analysis of recombinant human fumarase variants: lane 1) protein molecular mass standards, 2) HsFH, 3) A308T, and 4) H318Y. (**B**) Analysis of *E. coli* fumarase variants: lane 1) protein molecular mass standards, 2) FumC, and 3) E315Q (which is equivalent to E362Q in HsFH).

BN-PAGE experiments confirmed the oligomerization defects of the fumarase variants. In the non-denaturing gel, the majority of HsFH migrated with a molecular mass between that of the 242 and 480 kDa marker proteins (Fig. **6A**). Although HsFH migrates slightly larger than the calculated molecular mass for a tetramer, we assume the major band corresponds to the homotetramer since our analytical gel filtration results show that the bulk of HsFH forms a tetramer. Additionally, numerous prior studies have indicated that HsFH is a homotetramer [40, 49, 50]. A minority of HsFH migrated with a molecular mass consistent with it being an octamer, consistent with our gel filtration results and native PAGE analysis of *H. pylori* fumarase [65].

In contrast to HsFH, variants A308T and H318Y showed altered oligomerization states (Fig. **6A**). A majority of the variant proteins form dimers, as they appear to migrate as if they were half the size of HsFH. This result is consistent with the idea that the mutations within the intersubunit interface of the enzyme disrupt oligomerization. A small proportion of the variants appeared to form tetramers, migrating similarly to HsFH. As a control, we analyzed *E. coli* FumC and the FumC active site variant, E315Q, both of which crystallize as tetramers [3]. We found that FumC migrates near the 242 kDa marker (Fig. **6B**), again larger than its predicted tetrameric molecular mass of 205 kDa. In addition, the *E. coli* active site mutant E315Q, which corresponds to E362Q in HsFH, migrated similarly to FumC (Fig. **6B**). Thus, not all fumarase mutations disrupt oligomerization. Previously, E315Q was shown to directly perturb turnover number by diminishing the affinity for the active site water [3].

## DISCUSSION

**Fig. (7):**
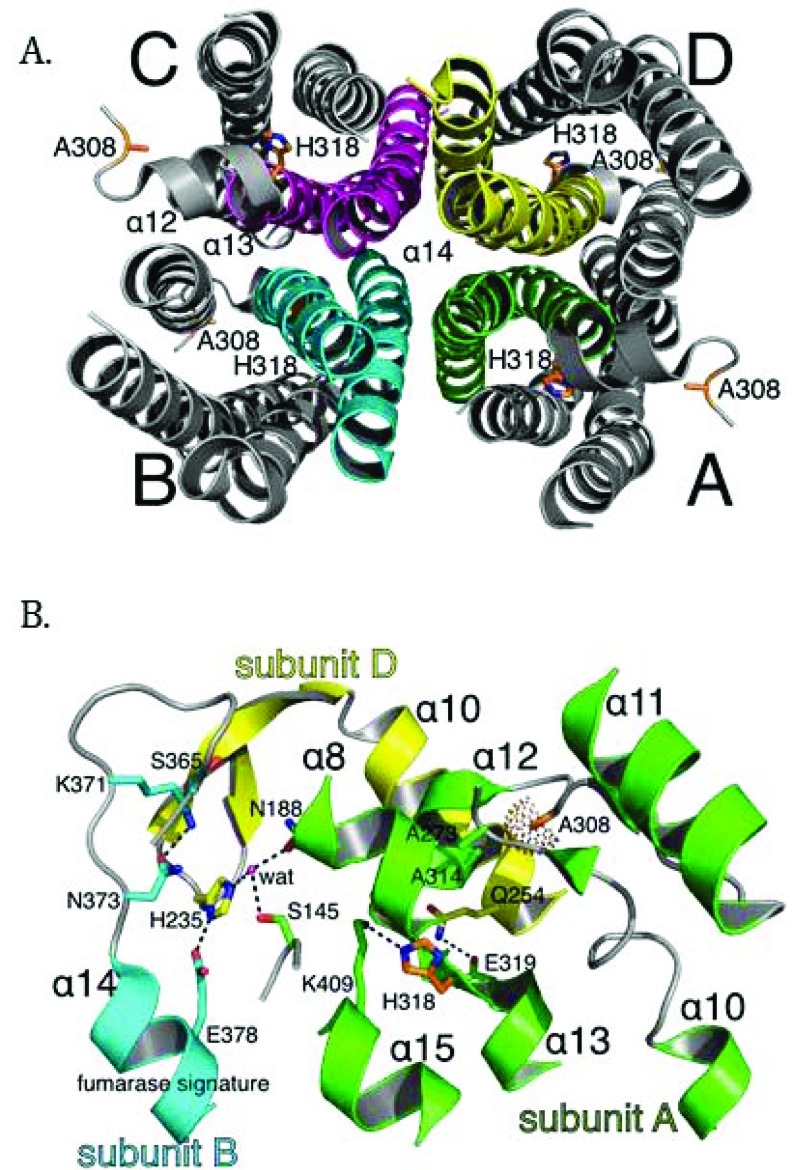
The putative structural impact of the A308T and H318Y variants at the multi-subunit active site in the human fumarase structure (PDB ID: 3E04 [40]). (**A**) The human fumarase tetramerization interface has been displayed looking down related D2 domains. For clarity, D1 and D3 have been removed from this image. Upon oligomerization, α-helix 14 lies at the center of the homotetramer. Additionally, α-helices 12 and 13 contribute residues to the tetramer interface. (**B**) The three-subunit fumarase active site has been depicted with emphasis upon the locations of A308 and H318 in context to the A-D dimer interface and fumarase signature sequence (GS^365^SxxPxK^371^xN^373^). Dashed lines depict hydrogen bonds and the dot surface reflects the A308 Cβ atom van der Waal radius. Residual monomer elements have been colored as depicted in Fig. (**1B**). The side chain atoms for A308 and H318 have been depicted in stick format and colored by atom type (red-oxygen, blue-nitrogen, orange-carbon). The fumarase active site water (wat) has been depicted as a magenta colored sphere.

Given our gel filtration and BN-PAGE results, we propose that both point mutants prevent stable HsFH tetramerization, and thereby disrupt formation of the active site leading to diminished catalytic function. Fumarase catalytic activity is inherently tied to homotetramer formation, as residues from three different subunits are required to assemble a functional active site [1, 43]. Structurally, A308 and H318 lie within regions forming intersubunit interactions, including α-helices 12 - 14 (Fig. **7A**). More specifically, A308 lies buried within a loop connecting α-helices 11 and 12. The buried region around A308 also includes A273 and A314 as donated by the loop between α-helix 10 and 11, and α-helix 12, respectively (Fig. **7B**). Therefore, the local region surrounding A308 is structurally restricted and incompatible with bulky side chain placement. Thus, threonine substitution at this location (A308T) would certainly require conformational adaptation to accommodate this β-branched and polar side chain. The gel filtration and BN-PAGE results support structural adaptation within the A308T variant via homodimer formation (Fig. **6A**).

The localization of H318, a polar/charged side chain, within the solvent inaccessible core of human fumarase is atypical. This atypical localization of H318 within the solvent inaccessible region of HsFH is electrostatically accommodated via an intrasubunit hydrogen bond with K409 (Fig. **7B**). Interestingly, D319 (one residue removed from H318 within α-helix 13) is also buried at the solvent inaccessible fumarase subunit interface. Alternatively, D319 is located at an intersubunit interface. D319 (subunit A) forms a hydrogen bond with Q254 (subunit D) to stabilize this dimer interface (Fig. **7B**). Thus, the intrasubunit hydrogen bond between H318 and K409 facilitates formation of the intersubunit hydrogen bond shared between Q254 and D319 as donated by α-helices 10 and 13 from subunits D and A, respectively. Structurally, the region occupied by H318 would be unable to accommodate the substitution of the larger tyrosine side chain. Ultimately, the replacement of histidine 318 with a tyrosine (H318Y) may impose structural consequences that disrupt both the H318:K409 intrasubunit and D319:Q254 intersubunit electrostatic interactions leading to destabilization of the 3,000 Å^2^ A-D intersubunit interface (Fig. **3A**). The gel filtration and BN-PAGE results for H318Y (Fig. **6A**) support this model.

How do the oligomerization defects fit with our kinetic results where A308T and H318Y have K_m_ values similar to HsFH yet diminished turnover numbers? Past investigations have suggested that regions from three of the four subunits are required for a functional fumarase active site [43, 47]. Therefore, we hypothesize that the residual A308T and H318Y activity (Table **1**) is due to the observed small fraction of homotetrameric species (Fig. **6A**). While the turnover rates of A308T and H318Y are >900-fold reduced compared to HsFH, their defects in tetramerization do not seem to be as profound. Thus, it is likely that the atomic-level alterations of the residue substitutions on protein structure, such as the disruption of significant electrostatic interactions described in the prior paragraph, have additional negative consequences on active site formation. In other words, although a small fraction of the mutant proteins can form tetramers (Fig. **6A**), the tetramers are not enzymatically active due to small structural changes that cannot be detected with gel filtration or BN-PAGE. Additional higher resolution structural studies are required to detect these subtle differences in structure.

We hypothesize the C-D dimer (Fig. **3B**) as the putative species observed during our gel filtration and BN-PAGE studies. We will use the amount of surface area in conjunction with the proximity of A308 and H318 to establish support for the C-D dimer as the experimentally observed dimer. According to PISA [62], the three dimer combinations ranked in order of decreasing buried surface area (Å^2^) are A-D, C-D, and B-D (Fig. **3**). Thus, the greatest amount of shared interface resides in the A-D dimer, followed by the C-D dimer and lastly the B-D dimer. The calculated A-D dimer interface is the most stable. However, the arrangement of the paired A308 and H318 side chains within each subunit align closest within the A-D dimer (Figs. **3A** and **7B**). Likewise, alteration of these residues would have the most impact on α-helices 12 and 13 in the A-D dimer. The B-D dimer has very little shared surface area and an average ΔG (P-value) of 0.522. A ΔG (P-value) of this magnitude suggests limited interaction specificity. Therefore, we hypothesize the C-D dimer as the species observed during our gel filtration and BN-PAGE studies. The C-D dimer has a related average ΔG (P-value) of 0.057, which indicates high specificity for this dimer interface [62]. Interestingly, the C-D dimer (symmetrical to the A-B dimer illustrated in (Fig. **7B**) contributes the two highly conserved regions (Fig. **2**) that build the substrate-binding site.

## CONCLUSION

In conclusion, our results indicate that recombinant human fumarase purified from *E. coli* is enzymatically active. Furthermore, we have shown that the A308T and H318Y variants are both enzymatically inactive and defective for oligomerization. Therefore, some forms of FHD and HLRCC may be linked to improperly folded quaternary structure.

## Figures and Tables

**Table 1:** Steady-state kinetic parameters of HsFH, A308T, and H318Y.

**Enzyme**	**V_max_ (µmole /min/mg)**	**k_cat_ (s^-1^)**	**K_m_ (mM)**	**k_cat_/K_m_ (M^-1^ s^-1^)**
HsFH	170 ± 10	150 ± 9	1.9 ± 0.2	8 x 10^4^ ± 1 x 10^4^
A308T	0.18 ± 0.02	0.16 ± 0.02	1.1 ± 0.3	150 ± 20
H318Y	0.046 ± 0.003	0.041 ± 0.002	2.0 ± 0.2	21 ± 2

Values represent the mean of four independent replicates ± standard deviation.
